# Change of Endoglucanase Activity and Rumen Microbial Community During Biodegradation of Cellulose Using Rumen Microbiota

**DOI:** 10.3389/fmicb.2020.603818

**Published:** 2020-12-18

**Authors:** Shuhei Takizawa, Ryoki Asano, Yasuhiro Fukuda, Mengjia Feng, Yasunori Baba, Kenichi Abe, Chika Tada, Yutaka Nakai

**Affiliations:** ^1^Laboratory of Sustainable Animal Environment, Graduate School of Agricultural Science, Tohoku University, Osaki, Japan; ^2^Japan Society for the Promotion of Science, Chiyoda-ku, Japan; ^3^Department of Agro-Food Science, Faculty of Agro-Food Science, Niigata Agro-Food University, Tainai, Japan; ^4^Research Institute for Bioresources and Biotechnology, Ishikawa Prefectural University, Nonoichi, Japan

**Keywords:** rumen microbial community, endoglucanase, zymogram, metagenomic sequencing, cellulose, lignocellulosic biomass

## Abstract

Treatment with rumen microorganisms improves the methane fermentation of undegradable lignocellulosic biomass; however, the role of endoglucanase in lignocellulose digestion remains unclear. This study was conducted to investigate endoglucanases contributing to cellulose degradation during treatment with rumen microorganisms, using carboxymethyl cellulose (CMC) as a substrate. The rate of CMC degradation increased for the first 24 h of treatment. Zymogram analysis revealed that endoglucanases of 52 and 53 kDa exhibited high enzyme activity for the first 12 h, whereas endoglucanases of 42, 50, and 101 kDa exhibited high enzyme activities from 12 to 24 h. This indicates that the activities of these five endoglucanases shifted and contributed to efficient CMC degradation. Metagenomic analysis revealed that the relative abundances of *Selenomonas*, *Eudiplodinium*, and *Metadinium* decreased after 12 h, which was positively correlated with the 52- and 53-kDa endoglucanases. Additionally, the relative abundances of *Porphyromonas*, *Didinium*, unclassified Bacteroidetes, *Clostridiales* family XI, *Lachnospiraceae* and *Sphingobacteriaceae* increased for the first 24 h, which was positively correlated with endoglucanases of 42, 50, and 101 kDa. This study suggests that uncharacterized and non-dominant microorganisms produce and/or contribute to activity of 40, 50, 52, 53, and 101 kDa endoglucanases, enhancing CMC degradation during treatment with rumen microorganisms.

## Introduction

Lignocellulosic biomass is the most abundant organic resource. The global annual production of lignocellulosic biomass is estimated to be approximately 181.5 billion tons, 4.6 billion tons of which are derived from agricultural residue (Dahmen et al., 2018). Lignocellulosic biomass has attracted attention recently for the production of bioenergy such as biogas, bioethanol, and bio-oil because bioconversion of lignocellulosic biomass does not lead to competition with food and feed. Lignocellulosic biomass is composed of cellulose, hemicellulose, and lignin. However, these polymers bind with each other and form a strong structure that resists disintegration and hydrolysis during anaerobic digestion (Sawatdeenarunat et al., 2015). To improve the bioconversion of lignocellulosic biomass, previous studies proposed physical (Rusanowska et al., 2018), chemical (Koyama et al., 2017), and biological (Dollhofer et al., 2018) pretreatments. These pretreatment methods enhance methane production from lignocellulosic biomass but consume large amounts of energy and wastewater neutralization and are costly. Baba et al. (2013) proposed pretreatment with rumen fluid as a novel method for efficient methane production from lignocellulosic biomass to overcome these limitations (Baba et al., 2013).

The rumen is an evolved forestomach that serves as one of the four stomachs of ruminants and drives microbial digestion of the ingested feed. The rumen fluid contains a complex microbial community consisting of bacteria, archaea, fungi, and protozoa. The rumen microbial community secretes various fibrolytic enzymes, such as endoglucanases (EC 3.2.1.4), exoglucanases (EC 3.2.1.91), beta-glucosidases (EC 3.2.1.21), xylanases (EC 3.2.1.8), and lignin peroxidase (EC 1.11.1.14), and efficiently converts lignocellulosic feed into volatile fatty acids (VFAs) that are major nutrient sources for the host animal (Kamra, 2005). Therefore, ruminant livestock can convert human-indigestible lignocellulose into readily accessible animal products using highly active fibrolytic enzymes of the rumen microbial community. In Japan, approximately 88,000 tons of rumen fluid are estimated to be discharged from slaughterhouses and currently treated as wastewater, the treatment of which consumes vast amounts of energy because of the high concentration of organic compounds in the rumen fluid. Applications with ruminal microorganisms are effective for degrading lignocellulosic biomass and are readily available from slaughterhouses. Pretreatment with rumen fluid has been shown to improve biogas production from agro-industrial and industrial paper waste (Baba et al., 2013, 2017; Takizawa et al., 2018) in methane fermentation. This technique also enhances VFA production from wheat straw, paten hay, lucerne hay, and corn silage (Nguyen et al., 2019).

Understanding the fibrolytic enzymes of ruminal microorganisms is essential for improving the bioconversion of lignocellulosic biomass. Endoglucanase (EC 3.2.1.4), which is one of three types of cellulases, can randomly cleave the internal beta-1,4-glycosidic bonds in amorphous regions of cellulose polymers. Endoglucanases in the rumen microbial community have been investigated by genetically analyzing ruminal microorganisms and through biochemical analysis of purified endoglucanases. Genetic analyses, such as metagenomics and meta-transcriptomics, have revealed the relative abundance of rRNA genes, functional genes, and transcripts encoding glycoside hydrolases in cellulolytic microorganisms of rumen microbial communities (Brulc et al., 2009; Dai et al., 2015; Lee et al., 2020). Furthermore, biochemical analyses of the crude enzymes demonstrated the characteristics of endoglucanases such as their enzymatic activity, molecular weight, optimal reaction pH, and optimal reaction temperature (Deguchi et al., 1991; Wood et al., 1995; Rincon et al., 2001). Additionally, enzyme assays of the rumen contents revealed the overall endoglucanase activity of different rumen microbial communities (Moon et al., 2010; Li et al., 2017). However, these previous investigations did not provide specific activities of individual endoglucanases in a complex microbial ecosystem; thus, the key endoglucanases that contribute to cellulose degradation during the treatment with rumen fluid are not well understood. This study was conducted to determine the temporal dynamics of individual endoglucanase activities during treatment of cellulose with rumen fluid, as well as endoglucanases that contribute to cellulose degradation by using a combination of chemical, enzymatic, and microbiological analyses. We determined the chemical characteristics of cellulose degradation during treatment with rumen fluid. Additionally, we conducted the zymogram analysis to visualize the enzyme activity of proteins after separation by sodium dodecyl sulfate-polyacrylamide gel electrophoresis (SDS-PAGE; Willis et al., 2010). Using metagenomic analysis, we detected the change in the structure and the metabolic characteristics of the rumen microbial community during treatment. Finally, we compared and found the relationship between the chemical, enzymatic, microbiological characteristics to reveal the endoglucanase and microorganisms related to cellulose degradation. This study reveals detailed information on the chemical, microbiological, and enzymatic characteristics of a complex rumen microbial community during treatment with rumen fluid, providing a foundation for the explanation of how the ruminal microbial community digests lignocellulosic biomass.

## Materials and Methods

### Treatment of Cellulose With Rumen Fluid

All experiments were approved and performed in accordance with the regulations of the Institutional Animal Care and Use Committee of Tohoku University. A lactating Holstein cow, whose body weight was 873 kg, was selected for the collection of rumen fluid. The diet consisted of 64% timothy grass and 36% concentrate, and the cow was allowed *ad libitum* access to water. Rumen fluid was collected at 2 h post-feeding using a stomach tube and filtered through a 1 mm × 1 mm mesh to remove coarse solids. The total solids (TS) content of the filtered rumen fluid was 5.3 g L^–1^.

Carboxymethyl cellulose (CMC) was used as a model substrate of cellulose because it can be degraded and solubilized by microorganisms producing endoglucanases. Four grams of CMC powder were mixed with 200 mL of rumen fluid and purged with nitrogen gas to remove oxygen. The initial pH of this mixture was 7.2 ± 0.1. Treatment of CMC was performed in a 250-mL reactor at 37°C for 48 h on a rotary shaker at 170 rpm. The blank reactor contained only rumen fluid, and its initial pH was 7.5 ± 0.1. Treatment of CMC with rumen fluid was conducted in duplicate.

### Chemical Analysis

To evaluate the rate of CMC conversion into VFAs and gases, TS, which included the total suspended solids and total dissolved solids, was measured after drying the rumen samples at 105°C (APHA, 2012). Next, reducing sugars (i.e., oligo- and mono-saccharides), VFAs (i.e., acetic acid, propionic acid, and butyric acid), and gas production (i.e., CH_4_ and CO_2_) were measured as described previously (Takizawa et al., 2018, 2019) to evaluate the metabolic production from CMC. Briefly, the gas concentration (CH_4_ and CO_2_) was measured by gas chromatography (GC-8A; Shimadzu, Kyoto, Japan). Liquid samples to be used for the analysis of VFAs and dissolved chemical oxygen demand (COD) were filtered using a cellulose acetate membrane filter (0.45 μm pore diameter). The VFA concentrations were determined by high-performance liquid chromatography (Jasco, Tokyo, Japan) using an ion-exchange column (RSpak KC-811; Shodex, Tokyo, Japan) and an ultraviolet detector (870-UV; JASCO). The dissolved COD, which indicates the dissolved organic compounds such as saccharides and VFAs, was determined using a colorimetric method with 0–1500 mg L^–1^ vials (Hach, Loveland, CO, United States). Reducing sugars were determined using the Somogyi–Nelson method (Nelson, 1944; Somogyi, 1945) on a UV–VIS spectrophotometer (Shimadzu). The pH was measured with a pH meter (LAQUAtwin; HORIBA, Kyoto, Japan).

### SDS-PAGE and Zymograms

Sodium dodecyl sulfate-polyacrylamide gel electrophoresis and zymogram analysis were performed according to a previous study (Takizawa et al., 2020). Briefly, 700 μL of rumen sample at 0, 6, 12, 24, and 48 h of the treatment was mixed with an equal volume of 2× sample buffer comprising 20% (v v^–1^) glycerol, 4% (w v^–1^) SDS, 0.01% (w v^–1^) bromophenol blue, 0.125 M Tris–HCl (pH 7.4), 10% (v v^–1^) 2-mercaptoethanol (pH 6.8), 2 mM phenylmethylsulfonyl fluoride, and 1× proteinase inhibitor. Protein extraction was carried out using two rounds of 2 min bead-beating with Lysing Matrix E tubes (MP Biomedicals, Santa Ana, CA, United States), followed by centrifugation at 12,000 *g* for 10 min. The supernatant was heated at 70°C for 20 min and immediately cooled on ice. Twenty microliters of protein extract were separated on an 8% polyacrylamide gel containing 0.15% (w v^–1^) CMC sodium salt (degree of substitution was from 0.5 to 0.8.). Electrophoresis was performed at 200 V for 60 min. Separated proteins were refolded in 20 mM phosphate-citrate buffer (pH 6.5) containing 1.5% (w v^–1^) β-cyclodextrin for 15 min. The gel was soaked in 50 mM sodium acetate buffer (pH 6.5) for 15 min, followed by incubation at 37°C for 90 min in 30 mM sodium acetate buffer. The pHs used for the zymogram during CMC treatment at 0, 6, 12, 24, and 48 h were 7.2, 7.1, 6.7, 5.5, and 5.3, respectively (see Supplementary Figure S1). To visualize the endoglucanase activity, the gel was stained with 0.1% Congo Red (w v^–1^) and de-stained with 1 M NaCl. To enhance the visualization of active bands, the gel was soaked in 0.3% (v v^–1^) acetic acid. Band densities were quantified with ImageJ software (NIH, Bethesda, MD, United States) (Schindelin et al., 2012).

### Real-Time PCR Amplification

Total DNA was extracted from 2 mL of rumen fluid using the FastDNA SPIN Kit for Soil (MP Biomedicals) according to the manufacturer’s instructions. Real-time PCR amplification was determined as described previously (Takizawa et al., 2020). In brief, the extracted DNA was amplified to quantify rumen microorganisms using primers for targeting general bacterial 16S rRNA genes (Forward, 5′-CGGCAACGAGCGCAACCC-3′; Reverse, 5′-CCATTGTAGCACGTGTGTAGCC-3′), anaerobic fungal 18S rRNA and ITS1 genes (Forward, 5′-GAGGAAGTAAAAGTCGTAACAAGGTTTC-3′; Reverse, 5′-CAAATTCACAAAGGGTAGGATGATT-3′) (Denman and McSweeney, 2006), and protozoal 18S rRNA genes (Forward, 5′-GCTTTCGWTGGTAGTGTATT-3′; Reverse, 5′-CTTGCCCTCYAATCGTWCT-3′) (Sylvester et al., 2004). Samples were assayed in triplicate in a 25-μL reaction mixture comprising 12.5 μL of MightyAmp for Real Time (TB Green Plus) (TaKaRa Bio, Shiga, Japan), 0.5 μL of 10 μM forward primer, 0.5 μL of 10 μM reverse primer, 1 μL template, and 10.5 μL sterile water. The real-time PCR conditions for general bacteria and anaerobic fungi were as follows: 98°C for 2 min, followed by 40 cycles of 98°C for 30 s, 60°C for 15 s, and 68°C for 30 s. The real-time PCR conditions for protozoa were as follows: 98°C for 4 min, followed by 45 cycles of 98°C for 30 s, 55°C for 30 s, 68°C for 30 s, and a final extension of 6 min at 72°C. All real-time PCR amplifications and detections were performed using a Thermal Cycler Dice real-time system (TaKaRa Bio).

A standard curve for bacteria, fungi, and protozoa was constructed by using plasmid DNA containing the inserts of bacterial 16S rRNA genes, anaerobic fungal 18S rRNA and ITS1 genes, and protozoal 18S rRNA genes. Target genes were amplified using aforementioned primers in a 25-μL reaction mixture comprising 0.125 μL of 5 units μL^–1^ Ex Taq (TaKaRa Bio), 2.5 μL of Ex Taq buffer (10×), 2 μL of 2.5 mM dNTP mixture, 0.25 μL of 10 μM forward primer, 0.25 μL of 10 μM reverse primer, 1 μL mixed DNA extracts, and 18.875 μL sterile water. The PCR conditions for general bacteria and anaerobic fungi were as follows: 94°C for 2 min, followed by 40 cycles of 94°C for 30 s, 60°C for 15 s, and 68°C for 1 min. The PCR conditions for protozoa were as follows: 94°C for 4 min, followed by 45 cycles of 94°C for 30 s, 55°C for 30 s, 68°C for 1 min, and a final extension of 6 min at 72°C. PCR products were electrophoresed on agarose gel to verify single product formation, and then purified using FastGene^TM^ Gel/PCR Extraction kit (NIPPON Genetics Co., Ltd, Tokyo, Japan) according to the manufacturer’s instructions. Purified PCR products were ligated to T-Vector pMD19 (Simple) (TaKaRa Bio). Constructed plasmids were transformed into High Efficiency DH5α Competent Cells (GMbiolab Co., Ltd, Taichung, Taiwan), and then incubated on Luria-Bertani agar plate containing 50 mg L^–1^ ampicillin at 37°C overnight. The colonies were picked up and cultivated in the Luria-Bertani liquid medium containing 50 mg L^–1^ ampicillin at 37°C overnight. After the incubation, the plasmid DNA was extracted using FastGene^TM^ Plasmid Mini Kit (NIPPON Genetics Co., Ltd) according to the manufacturer’s instructions. The concentration of the plasmid DNA was determined using a NanoDrop ND-1000 (Thermo Fisher Scientific, Waltham, MA, United States). Copy number of each plasmid was calculated using the molecular weight of nucleic acid and the length of the plasmid DNA.

### Metagenomic Sequencing

DNA extracts were purified using Agencourt AMPure XP (Beckman Coulter, Brea, CA, United States) and then quantified on a NanoDrop-1000 device (Thermo Fisher Scientific) and the Qubit^®^ dsDNA HS Assay Kit (Life Technologies, Carlsbad, CA, United States). DNA libraries were constructed using the QIAseq FX DNA Library Kit (Qiagen, Hilden, Germany) following the manufacturer’s protocol. Paired-end sequencing was performed on an Illumina HiSeq X instrument (Illumina, San Diego, CA, United States). Data obtained from the metagenomic sequencing are summarized in Supplementary Table 1.

Paired-end sequences were uploaded to the Metagenome Rapid Annotation using Subsystem Technology server version 4.0.3 (Meyer et al., 2008) for functional and taxonomic annotations. Hierarchical functional predictions were performed against the Kyoto Encyclopedia of Genes and Genomes Orthology (KO) database (E value of <10^–5^, identity cut-off of >60%, and alignment length of >30). To explore the structure of the rumen microbial community, rRNA sequences were identified by BLAST searching against the Silva SSU database (E value of <10^–5^, identity cut-off of >97%, and alignment length of >15 bp) on Metagenome Rapid Annotation using Subsystem Technology. Sequences are available at Metagenome Rapid Annotation using Subsystem Technology under the project accession mgp88002.

### Statistical Analysis

Multiple comparisons were performed according to the Tukey–Kramer method using R package multcomp, version 1.4-13. Statistical significance was declared at p values lower than 0.05, whereas *p* values lower than 0.10 were described as tendencies. Pearson’s correlation coefficient and significant levels were evaluated using the R package corrplot, version 0.84 to determine the relationships between the endoglucanase band density and relative abundance of ruminal microorganisms. Correlations with p values lower than 0.05 were considered as statistically significant, whereas *p* values lower than 0.10 were described as tendencies. Principal coordinate analysis based on Bray–Curtis dissimilarity and diversity analysis were performed using R package vegan, versions 2.0–5.0.

## Results and Discussion

### Carboxymethyl Cellulose Degradation During Treatment With Rumen Fluid

**FIGURE 1:**
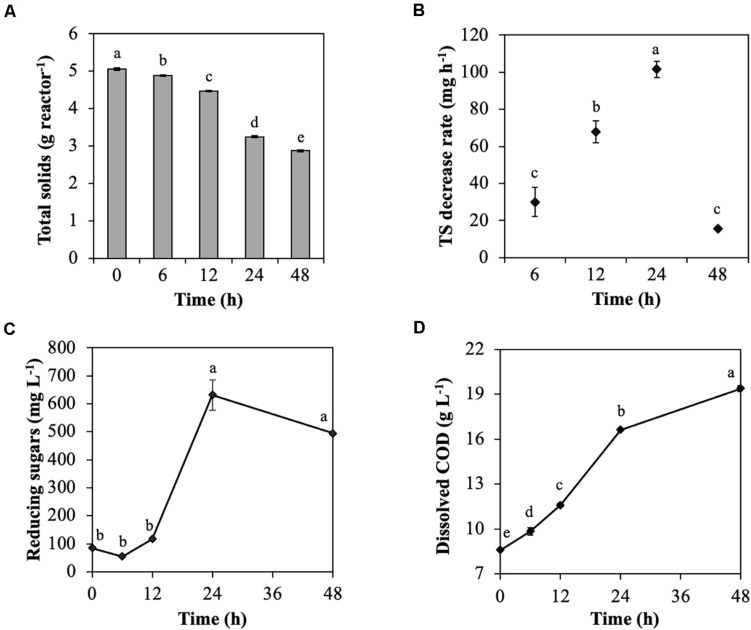
Characteristics of cellulose degradation during treatment with rumen fluid. **(A)** Total solids content; **(B)** total solids decrease rate; **(C)** reducing sugars; and **(D)** dissolved chemical oxygen demand (COD). Multiple comparisons were performed using the Tukey–Kramer method, and different letters indicate a statistically significant difference (*p* < 0.05).

The model substrate of cellulose, CMC, was degraded by the rumen microbial community during treatment for 48 h (Figure 1). TS was decreased throughout incubation, particularly from 24 to 48 h (from 4.5 to 3.3 g reactor^–1^; Figure 1A), which resulted in a considerable increase in the TS decrease rate from 12 to 24 h (from 68.0 to 101.6 mg h^–1^; Figure 1B). Reducing sugars that are produced during cellulose degradation significantly increased from 12 to 24 h (from 117.4 to 631.5 mg L^–1^; Figure 1C) due to CMC degradation by endoglucanases. Similarly, dissolved COD, which represent the solubilization efficiency of CMC, significantly increased from 12 to 24 h (from 11.6 to 16.6 mg L^–1^; Figure 1D). The dominant VFA was acetic acid, followed by propionic acid, butyric acid, valeric acid, and iso-valeric acid throughout the treatment of CMC with rumen fluid. Total VFAs increased throughout treatment of CMC, especially from 12 to 24 h (from 5.6 to 8.8 g L^–1^; Supplementary Figure 1A). The pH value reflected the total VFAs concentration and significantly decreased from 12 to 24 h (from 6.7 to 5.5; Supplementary Figure 1B). The volume of CH_4_, and CO_2_ production also increased from 12 to 24 h (Supplementary Figures 1C,D).

These results showed that the rumen microbial community efficiently digested CMC into cellodextrins, VFAs, and finally biogas (CO_2_ and CH_4_) from 12 to 24 h. Previous data showed that during biodegradation of rice straw with rumen fluid, cellulose degradability increased from 12 to 24 h, and VFAs and biogas were generated (Zhang et al., 2016), which is consistent with the results of the current study. Similarly, treating waste paper and rapeseed with rumen fluid resulted in high cellulose degradation from 6 to 24 h (Baba et al., 2013, 2017). In this study, it was expected that change in the characteristics of CMC degradation occurred because of the following two factors: (i) the increase in the total amount of endoglucanase and (ii) the change in endoglucanases with high enzyme activity. Denman and McSweeney (2006) reported that the populations of anaerobic fungi and cellulolytic bacteria *Fibrobacter succinogenes* increased in the rumen microbial community from 0 to 12 h after feeding of a basal diet of Angleton grass (Denman and McSweeney, 2006). Their reports support that the total amount of endoglucanases increased with the growth of microorganisms producing endoglucanases during CMC degradation, which increased endoglucanase activity. Additionally, Cheng et al. (2017) reported that the tightly rice straw-attached bacterial community shifted after 6 h of *in situ* incubation, resulting in decreases in *Anaerostipes*, *Blautia*, *Butyrivibrio*, *Coprococcus*, *Desulfovibrio*, *Prevotella*, *Pseudobutyrivibrio*, *Ruminococcus*, and *Succiniclasticum* and increases in *Clostridium*, *Dehalobacterium*, and *Oscillospira* (Cheng et al., 2017). Therefore, the shift in the rumen microbial community from microorganisms producing endoglucanases for the first 6 h to microorganisms producing endoglucanases with higher activity from 12 to 24 h, resulting in increased endoglucanase activity is expected.

The TS decrease rate of ruminal microorganisms declined after 24 h (Figure 1B). Ruminal pH values below 6.0 reduce adhesion and growth of cellulolytic bacteria, and ruminal pH values below 5.3 cease cellulose digestion because of lysis or detachment of adherent cellulolytic bacteria from undigested fiber (Mouriño et al., 2001). Major fibrolytic ruminal microorganisms, such as *Ruminococcus albus*, *Ruminococcus flavefaciens*, and *F. succinogenes*, are sensitive to acidity, and their growth is inhibited at pH values below 5.9 (Miwa et al., 1997). In this study, the effective conversion of CMC into VFAs resulted in VFAs accumulation and decreased pH, which may have inhibited the adherence and growth of cellulolytic microorganisms at 48 h.

### Temporal Dynamics of Endoglucanase Activity During Treatment With Rumen Fluid

**FIGURE 2:**
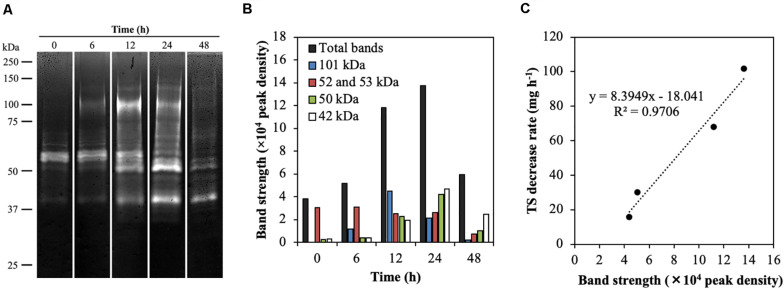
Endoglucanase activity in the rumen microbial community during CMC degradation. **(A)** Endoglucanase zymogram during CMC digestion. Twenty microliters of the protein extract were loaded on 8% polyacrylamide gel containing 0.15% (w v^−1^) CMC sodium salt, and the incubations for endoglucanase zymograms was performed at 37°C for 90 min. The pHs used for the zymogram in treatment of CMC at 0, 6, 12, 24, and 48 h were 7.2, 7.1, 6.7, 5.5, and 5.3, respectively (see Supplementary Figure S2). The grouping gels were cropped from different parts of the same gel. **(B)** Peak density of five endoglucanases having a high band intensity. **(C)** Correlation between the total band intensity of the five endoglucanases and total solids decrease rate.

To investigate the temporal dynamics of endoglucanase activity during treatment of CMC with rumen fluid, endoglucanase activity was detected by zymogram analysis under the pH conditions observed during treatment (Figure 2A and Supplementary Figure 2). In the blank (containing only rumen fluid), endoglucanases at 52 and 53 kDa showed high activity for the first 12 h, which then decreased (Supplementary Figure 3). The zymograms showed different banding patterns during the treatment of CMC with rumen fluid (Figure 2A). The total band densities increased for the first 24 h and then decreased (0 h, 54,608; 24 h, 208,838; 48 h, 49,594; Figure 2B). The time course of endoglucanase activity in this study is consistent with that reported previously for anaerobic fermentation of grass clippings by ruminal microorganisms for 72 h, which revealed that endoglucanase activity increased continuously during the first 24 h of fermentation and then began to decrease, with activity remaining stable after 48 h (Wang et al., 2018). The bands corresponding to 42, 50, 52, 53, and 101 kDa showed high endoglucanase activity (Figure 2B). The bands at 52 and 53 kDa showed high activity for the first 6 h, whereas the bands at 101, 50, and 42 kDa showed the highest activity at 12, 24, and 48 h, respectively. The bands at 52 and 53 kDa retained the activity from 0 to 12 h but decreased by 67% after 24 h. The band at 101 kDa showed 5-fold increased activity from 6 to 12 h, after which activity was decreased. The bands at 42 and 50 kDa showed increased activity by 8- and 12-fold from 6 to 24 h, and then the activity was remarkedly decreased. After 48 h, the bands at 52, 53, and 101 kDa became undetectable. These results reveal that the endoglucanase activities changed dynamically during treatment with rumen fluid. The total enzyme activity of the five endoglucanases of 42, 50, 52, 53, and 101 kDa increased when the TS decrease rate increased during treatment (*R*^2^ = 0.9477, *p* = 0.03, Figure 2C). Taken together, the CMC-degradation rate and temporal dynamics of endoglucanase activity indicate that the five endoglucanases of 42, 50, 52, 53, and 101 kDa play critical roles in CMC degradation during treatment with rumen fluid.

The decrease in endoglucanase activity at 48 h may have been due to the lowered pH (Supplementary Figure 1). Ruminal cellulolytic bacteria are known to cease growth at pH values below 5.9 (Miwa et al., 1997). Additionally, most fibrolytic enzymes secreted by ruminal microorganisms function optimally at pH values above 6.2 (Wang and McAllister, 2002). To investigate the effect of low pH on endoglucanase activity, protein extracts used in zymogram analysis as mentioned above were additionally applied to the zymogram at a pH of 6.5 (Supplementary Figure 4). The banding patterns of highly active endoglucanases at modulated pH 6.5 were similar to those observed without pH modulation, whereas more endoglucanases with low activities were detected from 35 to 250 kDa at modulated pH 6.5 than under conditions without pH modulation. These results indicate that decreasing the pH inhibited the activities of several endoglucanases (but not active endoglucanases) because of the shift in pH from their optimum pH. In addition, the low pH after 24 h possibly decreased the production of endoglucanases because of growth inhibition of cellulolytic microorganisms and/or the suppression of endoglucanase expression.

### Functional Characterization of Rumen Microbial Community

**FIGURE 3:**
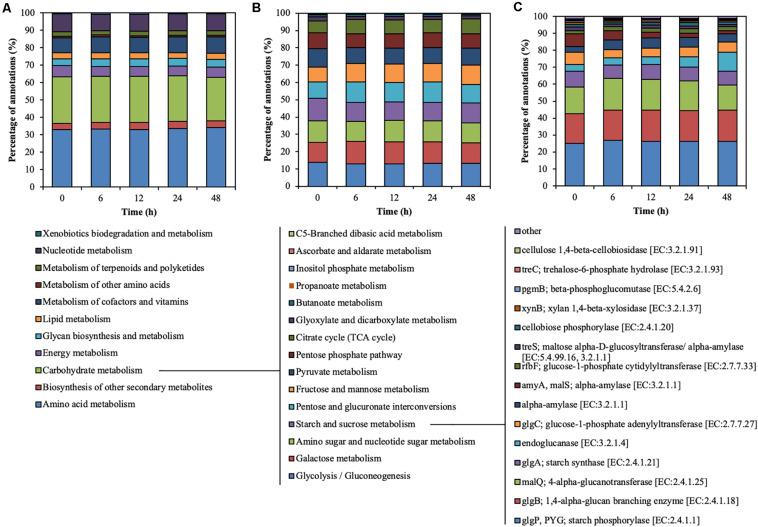
Hierarchical functional predictions for the rumen microbial community during treatment of CMC with rumen fluid. **(A)** Level 2 KO involved in metabolism; **(B)** level 3 KO involved in carbohydrate metabolism; and **(C)** functional KO involved in starch and sucrose metabolism. Data for the top 15 KO, and the other KO are included in the “other” category. Parameters for functional annotations were as follows; E value of <10^–5^, identity cut-off of >60%, and alignment length of >30. All values represent the mean of two reactors.

Metagenomic sequencing illustrated the functional characteristics of rumen microbial community during treatment of CMC with rumen fluid (Figure 3). Level-2 KO involved in carbohydrate metabolism slightly decreased from 0 to 6 h (from 26.89 to 26.29%) (*p* < 0.01) and from 24 to 48 h (from 26.29 to 24.94%) (*p* = 0.02) (Figure 3A). Level-3 KO involved in starch and sucrose metabolism slightly decreased from 0 to 6 h (from 12.94 to 10.81%) (*p* < 0.01) but increased from 24 to 48 h (from 10.67 to 11.53%) (*p* = 0.03) (Figure 3B). Functional KO annotated as endoglucanase [EC: 3.2.1.4] increased from 3.96 to 11.31% during CMC degradation, particularly from 24 to 48 h (from 5.77 to 11.31%) (*p* < 0.01) (Figure 3C). Similarly, the ratio of KO annotated as endoglucanases to total annotated KO was increased from 24 to 48 h (from 0.10 to 0.20%) (*p* < 0.01). These results demonstrate that the functional characteristics of endoglucanases in the rumen microbial community shifted during treatment with rumen fluid. Mayorga et al. (2016) reported that the bacterial function of the perennial ryegrass-attached microbiome differed between 1 and 4 h of colonization (Mayorga et al., 2016), which is consistent with the shifts in functional characteristics in this study. In this study, the TS decrease rate increased for the first 24 h and then decreased (Figure 1). Similarly, the endoglucanases of 42, 50, and 101 kDa increased for the first 24 h and decreased from 24 to 48 h (Figure 2). The ratio of KO annotated as endoglucanases increased from 24 to 48 h (Figure 3), which did not correlate with the CMC degradation rate or endoglucanase activity. These results indicate that the ratio of endoglucanase genes was not a key factor in determining the TS decrease rate and endoglucanase activity of 42, 50, and 101 kDa.

### Change in the Structure of the Rumen Microbial Community During Treatment

**FIGURE 4:**
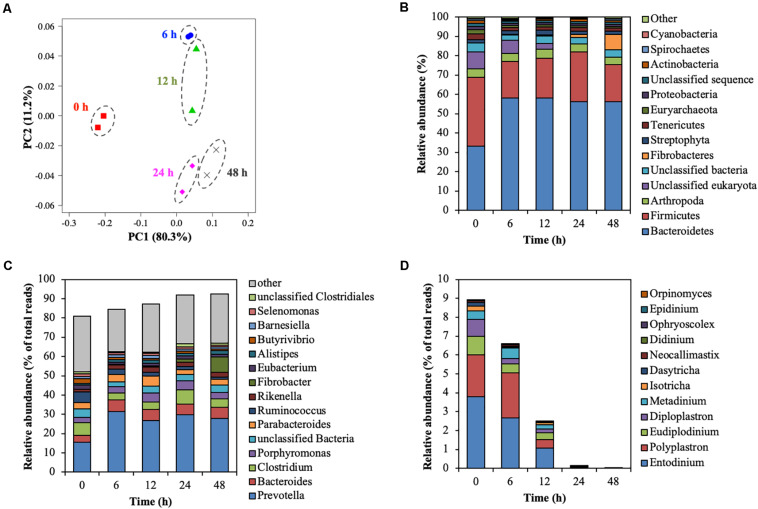
Shift of the rumen microbial community during treatment of CMC with rumen fluid. **(A)** Principal coordinate analysis (PCoA); **(B)** phylum-level analysis of the microbial community; **(C)** genus-level analysis of the bacterial community; and **(D)** genus-level analysis of the predominant protozoal and fungal community. Relative abundances of 28 of the 42 phyla were <0.5%, and these phyla were included in the “other” category. Data for the top 15 genus, and the other genera are included in the “other” category. Parameters for taxonomic annotations were as follows: E value of <10^–5^, identify cut-off of >97%, and alignment length of >15 bp. All values represent the mean of two reactors.

The rumen microbial community was analyzed by metagenomic sequencing. The diversity indices of the rumen microbial community decreased for the first 6 h, and then slightly recovered (Supplementary Table 2). Principal coordinate analysis illustrated the structure of the microbial community tended to cluster based on the treatment time (Figure 4A), which indicated the temporal changes in the microbial community during the treatment of CMC with rumen fluid. Twenty-five bacterial phyla were detected (Figure 4B). Bacteroidetes and Firmicutes were detected as the dominant bacterial phyla at 0 h; however, the relative abundance of Bacteroidetes increased from 33.35 to 58.12% of total reads after 6 h. These results are consistent with those of previous studies involving metagenomic sequencing, which showed that Bacteroidetes and Firmicutes were the dominant phyla during *in vivo* rumen fermentation (Brulc et al., 2009; Singh et al., 2012; Wallace et al., 2015) and that the relative abundance of Bacteroidetes increased during *in situ* incubation of switchgrass (Piao et al., 2014). Genus-level bacterial communities are shown in Figure 4C. In the initial bacterial community, the most dominant bacterial taxon was *Prevotella* (15.61% of total reads), followed by *Clostridium* (6.57% of total reads) and *Ruminococcus* (5.50% of total reads). The relative abundance of *Prevotella* and *Fibrobacter* significantly increased by 15.73% (from 15.61 to 31.34%) and 7.81% (from 0.16 to 7.96%) during treatment of CMC, respectively (*p* < 0.05). Meanwhile, the relative abundance of *Ruminococcus* and *Butyrivibrio* significantly decreased by 4.45% (from 5.50 to 1.06%) and 1.56% (from 2.39 to 0.83%), respectively (*p* < 0.05). These bacterial genera (*Prevotella*, *Clostridium*, *Fibrobacter*, *Ruminococcus*, and *Butyrivibrio*) include known cellulose-degrading bacterial species (Kamra, 2005), of which *Fibrobacter* and *Ruminococcus* are considered as the predominant cellulose degraders in the rumen (Dai et al., 2015; Baba et al., 2017). The degradation of rapeseed (*Brassica napus* L.) with rumen fluid resulted in an increased abundance of *Ruminococcus*, *Clostridium*, and *Butyrivibrio*, whereas the abundance of *Prevotella* and *Fibrobacter* decreased during incubation (Baba et al., 2017), which is not consistent with our current findings. This may have been caused by differences in the substrate composition. In this study, it was inferred that the relative abundances of *Prevotella* and *Fibrobacter* increased during treatment of CMC with rumen fluid because these genera efficiently utilized CMC and/or metabolic products for their growth. Genus-level protozoal and fungal communities are shown in Figure 4D. In the initial protozoal community, the most dominant protozoal genus was *Entodinium* (3.78% of total reads), followed by *Polyplastron* (2.23% of total reads), *Eudiplodinium* (0.99% of total reads), *Diploplastron* (0.90% of total reads), and *Metadinium* (0.43% of total reads). These predominant protozoal genera showed a significant decrease in their relative abundance and the relative abundance of eukaryotic genera significantly decreased from 8.78 to 0.02% of total reads during the treatment of CMC (*p* = 0.03). In the initial fungal community, the most dominant fungal genus was *Neocallimastix* (0.09% of total reads), followed by *Orpinomyces* (<0.01% of total reads). These fungal genera also showed a tendency to decrease their relative abundances from 0.09 to 0.00% of total reads during the treatment of CMC (*p* = 0.09). Our results showed the temporal dynamics of rumen microbial community during the treatment of CMC with rumen fluid. Rumen microbial abundances were quantified by real-time PCR (Supplementary Figure 5). The number of bacteria slightly increased from 10.3 to 10.9 log_10_ copies mL^–1^ during the treatment of CMC (Supplementary Figure 5A). Meanwhile, the fungal abundance decreased from 24 to 48 h (from 6.7 to 5.4 log_10_ copies mL^–1^) (Supplementary Figure 5B), and the protozoal abundance decreased from 12 to 48 h (from 8.7 to 5.5 log_10_ copies mL^–1^) (Supplementary Figure 5C). These results indicate that the growth and survival rates of fungi and protozoa were lower than those of bacteria during treatment with rumen fluid.

The results of endoglucanase zymogram and metagenomic sequencing indicated that temporal shifts in the relative abundance of cellulolytic microorganisms altered the temporal dynamics of the endoglucanase activity at 42, 50, 52, 53, and 101 kDa in size during treatment of CMC with the rumen fluid.

### Relationship Between Endoglucanase Activity and Microbial Abundance

**FIGURE 5:**
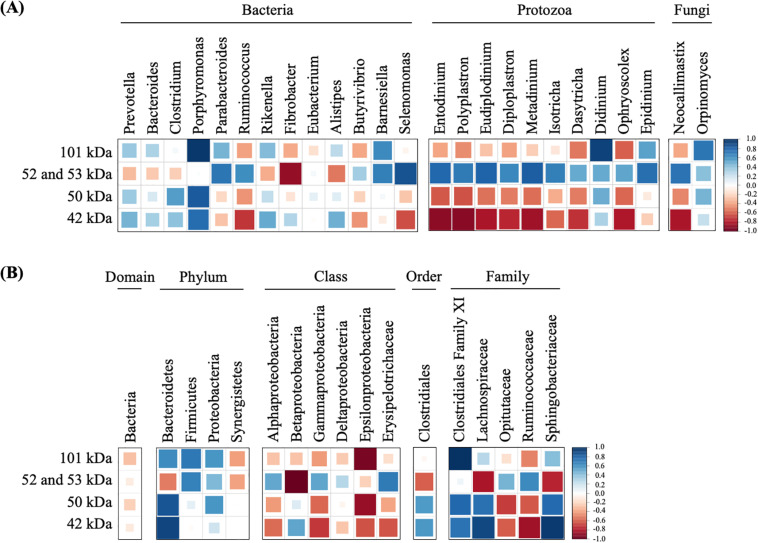
Correlations of dominant microorganisms **(A)** and unclassified microorganisms **(B)** with the band densities of highly active endoglucanases. The color and size of square represent each correlation coefficient. Blue shading represents a positive correlation, and red shading represents a negative correlation. A larger square represents a higher correlation, and a smaller square represents a weaker correlation.

To predict which microorganisms contributed to endoglucanase activity, the correlations between the band densities of major endoglucanases and relative abundances of ruminal microorganisms were analyzed (Figure 5 and Table 1). Predominant cellulolytic bacteria, such as *Prevotella*, *Bacteroides*, *Clostridium*, *Ruminococcus*, *Fibrobacter*, and *Butyrivibrio* showed no significant positive correlations with endoglucanase activities at 42, 50, 52, 53, and 101 kDa (*p* > 0.10, Table 1). Many previous studies showed that *Ruminococcus* and *Fibrobacter* produce cellulases and play key roles in cellulose degradation by the rumen microbial community (Dai et al., 2015; Baba et al., 2017). Most cellulolytic eukaryotes, such as *Entodinium*, *Polyplastron*, *Diploplastron*, *Neocallimastix*, and *Epidinium*, showed positive correlations with the endoglucanase activities of 52 and 53 kDa (*r* > 0.70); however, the correlations were not significant (*p* > 0.10). *Eudiplodinium* and *Metadinium* tended to show a positive correlation with the endoglucanases of 52 and 53 kDa (*r* > 0.80, *p* < 0.10). It has been reported that *Eudiplodinium maggii* secretes an endoglucanase of approximately 54 kDa (Béra-Maillet et al., 2005) and *Metadinium* exhibited cellulose degradation capacity in the rumen with high abundance (Langda et al., 2020); however, the contribution of *Eudiplodinium* and *Metadinium* to CMC degradation during treatment with rumen fluid is not well-understood.

**TABLE 1:** Relative abundance of cellulolytic microorganisms and their positive correlation with endoglucanase activity.

	**Relative abundance (% of total reads)**					**Positive correlation^†^**
		
**Genus**	**0 h**	**6 h**	**12 h**	**24 h**	**48 h**	
**Bacteria^††^**						
*Prevotella*	15.61^b^	31.34^a^	26.84^a^	29.94^a^	27.83^a^	-
*Bacteroides*	3.41^a^	6.16^a^	5.67^a^	5.40^a^	5.83^a^	-
*Clostridium*	6.57^b^	3.45^d^	3.86^cd^	7.36^a^	4.43^c^	-
*Ruminococcus*	5.50^a^	2.67^b^	1.98^bc^	1.56^bc^	1.06^c^	-
*Fibrobacter*	0.16^b^	0.28^b^	0.62^b^	1.51^b^	7.96^a^	-
*Eubacterium*	2.23^a^	1.03^b^	1.37^b^	1.72^ab^	1.38^b^	-
*Alistipes*	0.83^a^	1.40^a^	1.71^a^	1.41^a^	1.93^a^	-
*Butyrivibrio*	2.39^a^	0.96^b^	0.91^b^	1.18^b^	0.83^b^	-
*Capnocytophaga*	0.51^a^	0.92^a^	1.02^a^	0.77^a^	1.10^a^	-
*Tenacibaculum*	0.41^a^	0.73^a^	1.17^a^	0.70^a^	1.04^a^	-
*Paraprevotella*	0.37^a^	1.01^a^	0.93^a^	0.76^a^	0.64^a^	-
*Bacillus*	1.29^a^	0.57^b^	0.59^b^	0.72^b^	0.51^b^	-
*Pedobacter*	0.27^c^	0.44^bc^	0.86^a^	0.70^ab^	0.92^a^	-
*Kordia*	0.25^a^	0.62^a^	0.56^a^	0.66^a^	0.68^a^	-
*Sphingobacterium*	0.26^b^	0.46^ab^	0.60^a^	0.56^ab^	0.70^a^	-
**Protozoa**						
*Entodinium*	3.78^a^	2.90^a^	1.37^ab^	0.01^b^	0.01^b^	-
*Polyplastron*	2.23^ab^	2.56^a^	0.54^ab^	0.02^b^	0.00^b^	-
*Eudiplodinium*	0.99^a^	0.52^a^	0.46^a^	0.00^a^	0.00^a^	52 and 53 kDa
*Diploplastron*	0.90^a^	0.29^a^	0.23^a^	0.00^a^	0.00^a^	-
*Metadinium*	0.43^a^	0.61^ab^	0.26^ab^	0.04^b^	0.00^b^	52 and 53 kDa
*Epidinium*	0.00^a^	0.01^a^	0.02^a^	0.00^a^	0.00^a^	-
**Fungi**						
*Neocallimastix*	0.09^a^	0.07^a^	0.02^a^	0.02^a^	0.00^a^	52 and 53 kDa
*Orpinomyces*	0.00^a^	0.00^ab^	0.00^b^	0.00^b^	0.00^b^	-

*Cellulose-degrading and/or endoglucanase-producing microorganisms have been shown. Different letters indicate a statistically significant difference (*p* < 0.05). ^†^Endoglucanases with high enzyme activity which had a positive correlation to the relative abundance of the genus. ^††^Top 15 of cellulolytic bacteria were shown.*

Non-cellulolytic microorganism *Porphyromonas* showed a positive correlation with the endoglucanase of 50 kDa (*r* = 0.83, *p* = 0.08) and 101 kDa (*r* = 0.97, *p* = 0.01), and *Selenomonas* tended to show a positive correlation with the endoglucanases of 52 and 53 kDa (*r* = 0.87, *p* = 0.06, Figure 5A). Summanen et al. (2005) found that *Porphyromonas* fermented soluble sugars such as glucose, lactose, and mannose (Summanen et al., 2005); however, no cellulase genes were detected in the whole-genome data from *Porphyromonas gingivalis* strain ATCC33277 (Naito et al., 2008) and W83 (Nelson et al., 2003). Similarly, Prins (1971) revealed that *Selenomonas* could not hydrolyze cellulose but utilized soluble sugars (Prins, 1971). Therefore, *Porphyromonas* and *Selenomonas* may utilize CMC degradation products for their growth, resulting in the positive correlation with the endoglucanases of 50 and 101 kDa. In addition, *Didinium* showed a significant positive correlation with the endoglucanases of 101 kDa (*r* = 0.92, *p* = 0.03). *Didinium* is a protozoan predator but its cellulolytic activity has not been reported, indicating that *Didinium* indirectly contributed to CMC degradation by the predation of other protozoa, which engulf fibrolytic microorganisms. These results suggest that non-cellulolytic microorganisms do not produce endoglucanases with high activity but indirectly contribute to endoglucanase activities and play important roles in CMC degradation during treatment with rumen microbial community.

Interestingly, unclassified Bacteroidetes, unclassified *Clostridiales* family XI, unclassified *Lachnospiraceae*, and unclassified *Sphingobacteriaceae* showed significant positive correlations with the endoglucanases of 42, 50, and 101 kDa (*r* > 0.80, *p* < 0.05, Figure 5B). Although unclassified *Clostridiales*, unclassified *Lachnospiraceae*, and unclassified *Sphingobacteriaceae* are frequently detected in the rumen (Cheng et al., 2017; Yang et al., 2018), their biological characteristics were not known. Our results indicate that these uncharacterized microorganisms contributed to producing endoglucanases which showed high enzyme activity after 12 h. Approximately 77% of fiber-associated rumen microorganisms are uncultured and show less than 97% similarity with known isolates (Koike et al., 2003). Previous 16S rRNA-based analysis suggested that uncultured bacteria are responsible for fiber digestion in the rumen (Koike and Kobayashi, 2009). Metatranscriptomic analysis of bovine ruminal microorganisms has also suggested that most putative proteins involved in the degradation of plant cell wall polysaccharides are the unknown relatives of known genera or species with predominant roles in cellulose degradation in the rumen (Dai et al., 2015; Comtet-Marre et al., 2017). Taken together, our results suggest that *Eudiplodinium*, *Metadinium*, *Didinium*, unclassified Bacteroidetes, unclassified *Clostridiales* family XI, unclassified *Lachnospiraceae*, and unclassified *Sphingobacteriaceae* produced and/or contributed to the endoglucanase activities and accelerated the increase in CMC degradation by the rumen microbial community. This is the first study to propose that these uncharacterized microorganisms with low abundance contribute to endoglucanase activities and CMC degradation during treatment with rumen fluid. Our findings may be applied to improve methane production from lignocellulosic biomass by activating the rumen microorganisms contributing to endoglucanase activities. Further analysis and cultivation of these unclassified microorganisms should reveal whether these microorganisms produce and/or contribute to endoglucanase activities in the rumen microbial community during treatment with rumen fluid.

In conclusion, time-course experiments showed that the TS decrease rate was consistent with the endoglucanase activities. Zymogram revealed that endoglucanases of 52 and 53 kDa possessed the high band strength for the first 12 h, whereas endoglucanases of 42, 50, and 101 kDa showed high enzyme activities from 12 to 24 h. This finding indicate that endoglucanase activities shifted and contributed to CMC degradation during treatment with rumen fluid. Relative abundances of uncharacterized and non-dominant microorganisms were positively correlated with endoglucanases. This work reveals the temporal dynamics of endoglucanase activity and the contribution endoglucanases make to cellulose digestion in the rumen microbial community during treatment with rumen fluid. Further study using various lignocellulosic biomasses will identify these key endoglucanases and microorganisms, thereby providing insights into the mechanisms underlying lignocellulose degradation in the rumen microbial community. In addition, our findings indicate that activation of key endoglucanases and microorganisms is an efficient strategy to improve the bioconversion of lignocellulosic biomass into bioenergy.

## Data Availability Statement

The datasets presented in this study can be found in online repositories. The names of the repository/repositories and accession number(s) can be found in the article/Supplementary Material.

## Ethics Statement

The animal study was reviewed and approved by Institutional Animal Care and Use Committee of Tohoku University.

## Author Contributions

ST wrote the main manuscript text, prepared all figures and tables, and performed the chemical and protein analysis. RA, YF, YB, KA, CT, and YN designed and oversaw the project. ST and RA performed the metagenomic analysis. RA, YF, MF, and YB assisted with data analysis. All authors reviewed and assisted with the writing manuscript.

## Conflict of Interest

The authors declare that the research was conducted in the absence of any commercial or financial relationships that could be construed as a potential conflict of interest.
