# Supplementary material for: Change of Endoglucanase Activity and Rumen Microbial Community During Biodegradation of Cellulose Using Rumen Microbiota
Source: Front Microbiol. 2020 Dec 18;11:603818. doi: 10.3389/fmicb.2020.603818 (PMC7775302; doi:10.3389/fmicb.2020.603818)
Supplement: Supplementary file 1 [file Data_Sheet_1.pdf]

RESEARCH ARTICLE

Change of Endoglucanase Activity and Rumen Microbial Community During Biodegradation of Cellulose Using Rumen Microbiota

Shuhei Takizawa[1,2], Ryoki Asano[3], Yasuhiro Fukuda[1], Mengjia Feng[1], Yasunori Baba[4], Kenichi Abe[3], Chika Tada[1*], Yutaka Nakai[3**]

[1] Laboratory of Sustainable Animal Environment, Graduate School of Agricultural Science, Tohoku University, Osaki, Japan

[2] Research Fellow of Japan Society for the Promotion of Science, Japan Society for the Promotion of Science, Chiyoda-ku, Japan

[3] Department of Agro-Food Science, Faculty of Agro-Food Science, Niigata Agro-Food University, Tainai, Japan

[4] Research Institute for Bioresources and Biotechnology, Ishikawa Prefectural University, Nonoichi, Japan

Corresponding authors

***Chika Tada**: Laboratory of Sustainable Animal Environment, Graduate School of Agricultural Science, Tohoku University, Yomogida 232-3, Naruko-onsen, Osaki, Miyagi 989-6711, Japan. Tel: +81-229-84-7391, Fax: +81-229-84-7391, E-mail: chika.tada.e1@tohoku.ac.jp

****Yutaka Nakai**: Department of Agro-Food Science, Faculty of Agro-Food Science, Niigata Agro-Food University, Hiranedai 2416, Tainai, Niigata 959-2702, Japan. Tel: +81-254-28-9855, Fax: +81-254-28-9855, E-mail: yutaka-nakai@nafu.ac.jp

Supplementary data

Table caption

Table S1. Results of metagenomic sequencing analysis.

Table S2. Diversity indices of rumen microbial community during treatment of CMC with rumen fluid.

Figure legends

Figure S1. Time course of pH, total VFAs, CH_4 gas production, and CO_2 gas production during the treatment of CMC with rumen fluid.

Figure S2. The original zymogram gel before adjustments of contrasts and exposures shown in Fig. 2A. Twenty microliters of protein extract were loaded on 8% polyacrylamide gel containing 0.15%(w v^{-1}) CMC sodium salt, and the incubations for endoglucanase zymograms was performed at 37 ºC for 90 min. The pHs used for the zymogram at 0, 6, 12, 24, and 48 h were 7.2, 7.1, 6.7, 5.5, and 5.3, respectively. The grouping gels were cropped from different parts of the same gel.

Figure S3. Endoglucanase zymogram gels in the blank containing only rumen fluid. (A), the zymogram gel after adjustments of contrasts and exposures; and **(B)** the original zymogram gel. Twenty microliters of protein extract were loaded on 8% polyacrylamide gel containing 0.15%(w v^{-1}) CMC sodium salt, and the incubations for endoglucanase zymograms was performed at 37 ºC for 90 min. The pHs used for the zymogram were at pH 7.0 which was the average pH in the blank. The grouping gels were cropped from different parts of the same gel.

Figure S4. Endoglucanase zymogram gels under neutral pH conditions in the treatment of CMC with rumen fluid. (A), the zymogram gel after adjustments of contrasts and exposures; and **(B),** the original gel. Twenty microliters of protein extract were loaded on 8% polyacrylamide gel containing 0.15%(w v^{-1}) CMC sodium salt, and the incubations for endoglucanase zymograms was performed at 37 ºC for 90 min. The pHs used for the zymogram were at pH 6.5. The grouping gels were cropped from different parts of the same gel.

Figure S5. Change in the microbial abundance during treatment of CMC with rumen fluid. (A), bacterial abundance; **(B)**, fungal abundance; and **(C)**, protozoal abundance. Results are given as logarithms of copy numbers per 1 mL of rumen fluid ± standard error. Different alphabets indicate a significant difference ($p < 0.05$).

Table S1. Results of metagenomic sequencing analysis

	0 h	6 h	12 h	24 h	48 h
Total reads (bp)	1 949 158 266	1 473 774 524	1 483 675 694	1 881 185 793	1 827 125 603
Sequences count	9 115 530	6 948 679	7 106 036	8 693 400	8 595 123
Sequence length (bp)	214	211	209	217	213
Predicted protein features	3 036 986	2 324 200	2 384 873	2 886 501	2 865 836
Predicted rRNA features	11 198	8 618	8 595	9 420	8 826

Table S3. Diversity indices of rumen microbial community during treatment of CMC with rumen fluid

	0 h	6 h	12 h	24 h	48 h
Chao 1	447.338	383.937	375.007	350.969	373.647
ACE	434.075	358.647	368.488	345.428	386.814
Shannon index	3.802	3.177	3.359	3.307	3.359
Simpson index	0.939	0.846	0.885	0.876	0.888

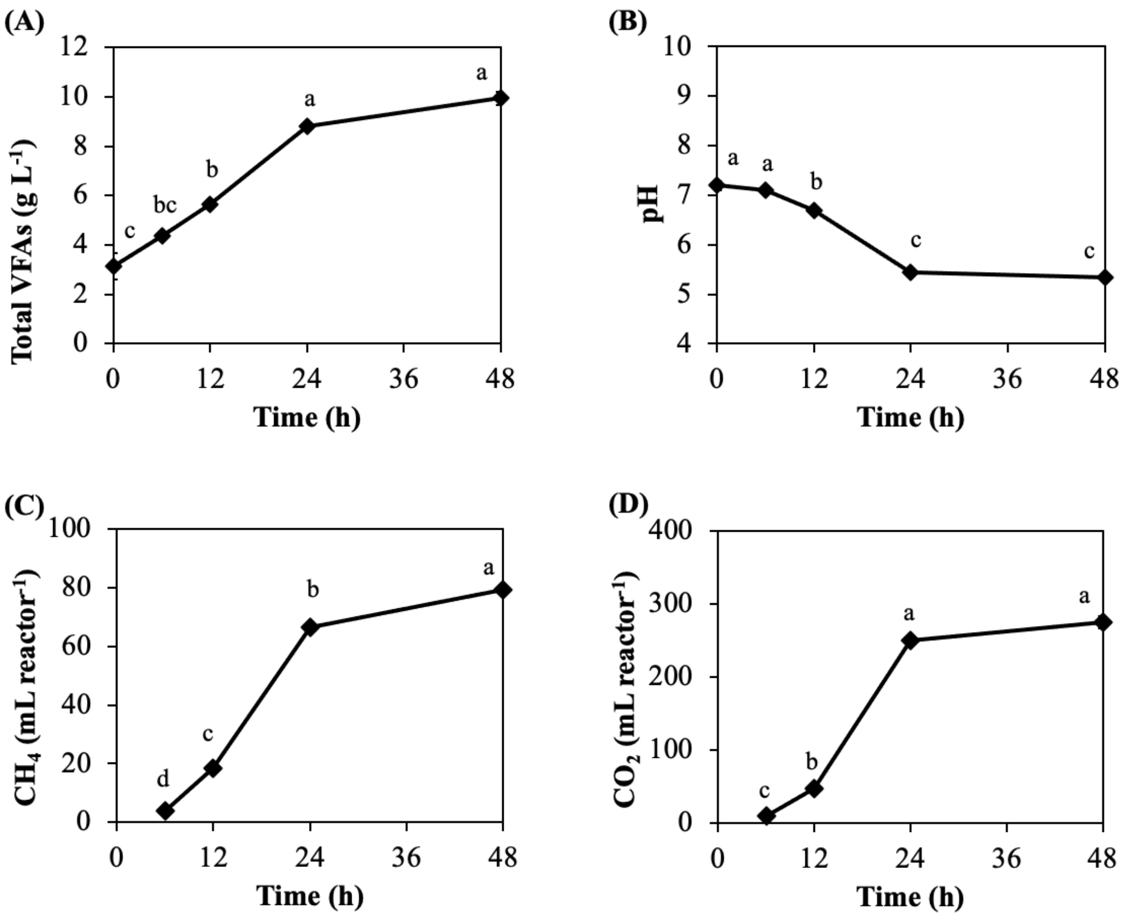

Figure S1. **Time course of pH, total VFAs, CH_4 gas production, and CO_2 gas production during the treatment of CMC with rumen fluid.** Multiple comparisons were performed using the Tukey-Kramer method, and different letters indicate a statistically significant difference ($p < 0.05$).

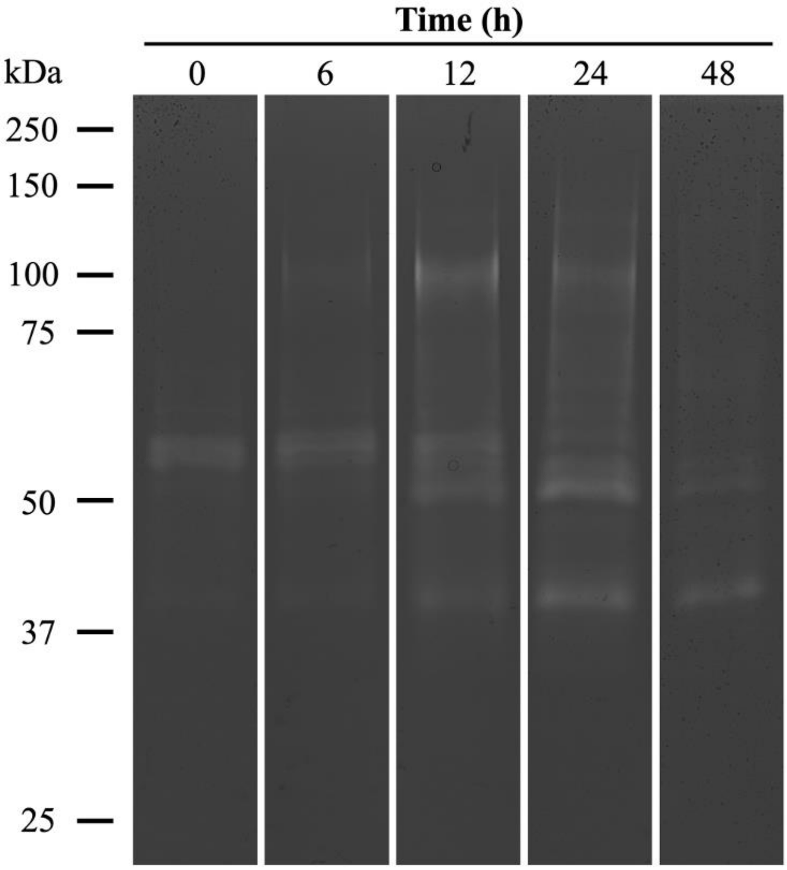

Figure S2. **The original zymogram gel before adjustments of contrasts and exposures shown in Fig. 2A.** Twenty microliters of protein extract were loaded on 8% polyacrylamide gel containing 0.15%(w v^{-1}) CMC sodium salt, and the incubations for endoglucanase zymograms was performed at 37 ºC for 90 min. The pHs used for the zymogram at 0, 6, 12, 24, and 48 h were 7.2, 7.1, 6.7, 5.5, and 5.3, respectively. The grouping gels were cropped from different parts of the same gel.

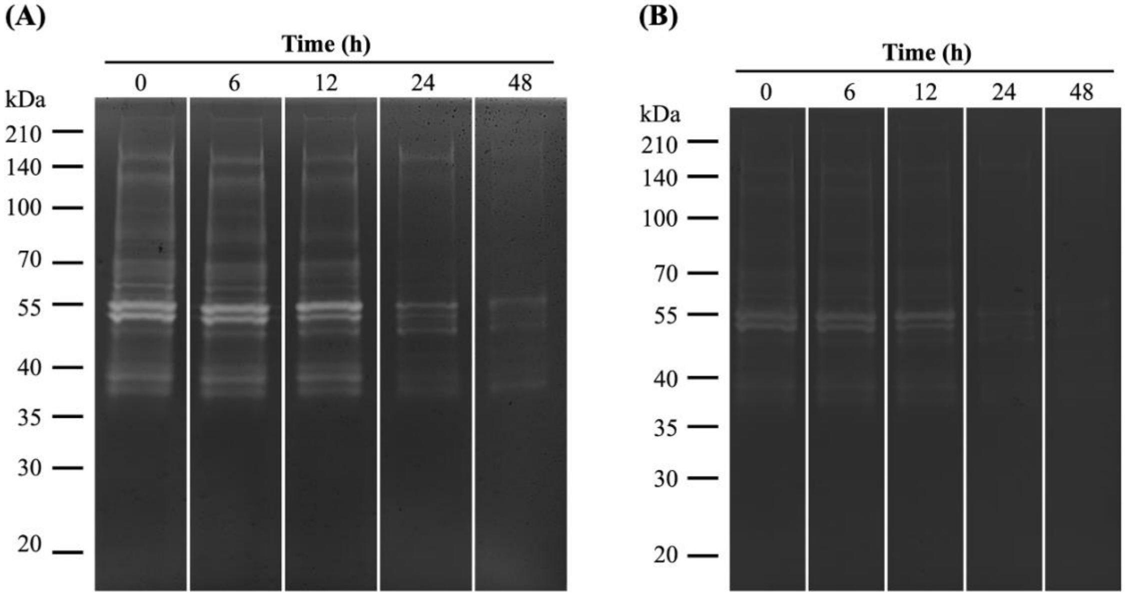

Figure S3. Endoglucanase zymogram gels in the blank containing only rumen fluid. (A), the zymogram gel after adjustments of contrasts and exposures; and **(B)** the original zymogram gel. Twenty microliters of protein extract were loaded on 8% polyacrylamide gel containing 0.15%(w v^{-1}) CMC sodium salt, and the incubations for endoglucanase zymograms was performed at 37 ºC for 90 min. The pHs used for the zymogram were at pH 7.0 which was the average pH in the blank. The grouping gels were cropped from different parts of the same gel.

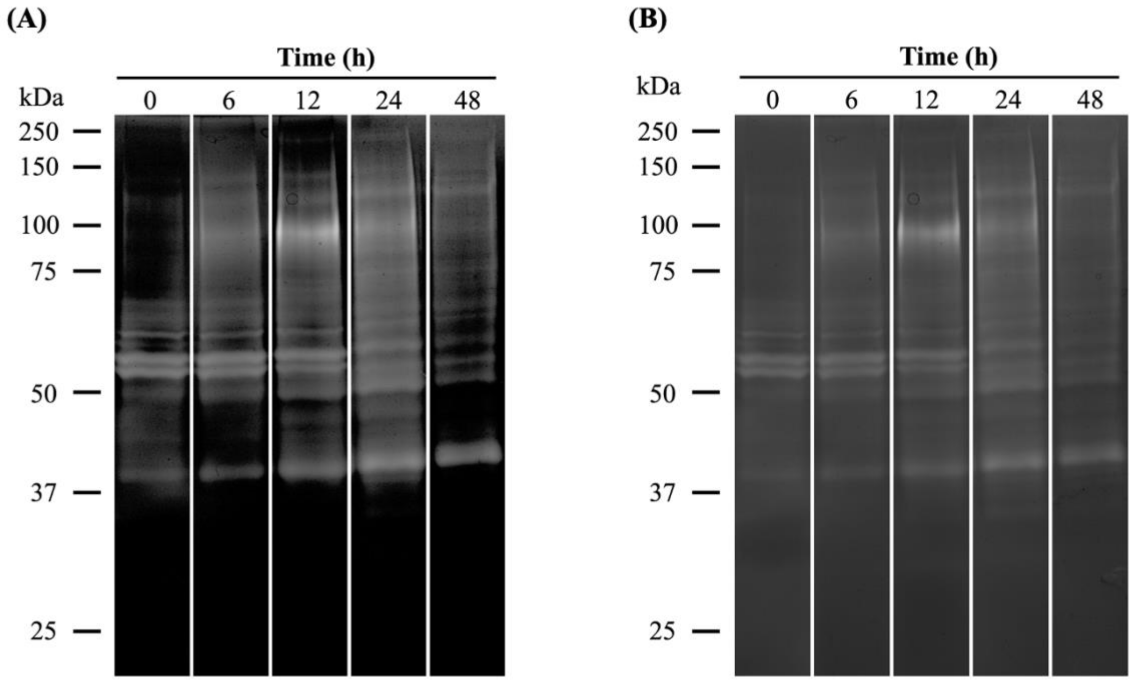

Figure S4. Endoglucanase zymogram gels under neutral pH conditions in the treatment of CMC with rumen fluid. (A), the zymogram gel after adjustments of contrasts and exposures; and **(B)**, the original gel. Twenty microliters of protein extract were loaded on 8% polyacrylamide gel containing 0.15%(w v^{-1}) CMC sodium salt, and the incubations for endoglucanase zymograms was performed at 37 ºC for 90 min. The pHs used for the zymogram were at pH 6.5. The grouping gels were cropped from different parts of the same gel.

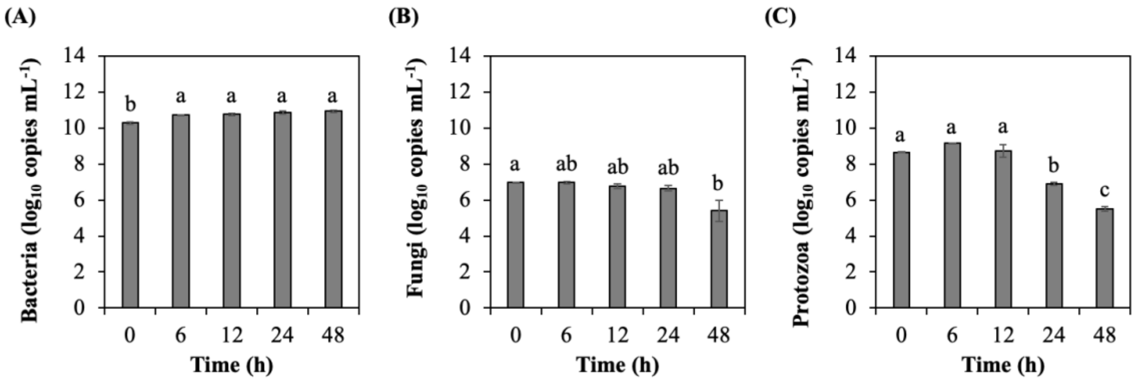

Figure S5. Change in the microbial abundance during treatment of CMC with rumen fluid. (A), bacterial abundance; **(B)**, fungal abundance; and (**C)**, protozoal abundance. Results are given as logarithms of copy numbers per 1 mL of rumen fluid ± standard error. Multiple comparisons were performed using the Tukey-Kramer method, and different letters indicate a statistically significant difference ($p < 0.05$).
